# Diseases of Circulatory System

**Published:** 1905-03-25

**Authors:** 


### DISEASES OF CIRCULATORY SYSTEM.

(*Continued from page* 447.)

**Bradycardia.**—G. A. Gibson [10] classifies the causes of bradycardia as nervous, toxic, or cardiac changes such as myocarditis, and failure of the auricular systole to pass on to the ventricle, so that the latter shows only one beat for two of the former. The condition may be persistent or paroxysmal, the latter being probably due to changes in the cerebral vessels, which produce brain anæmia and irritation of the inhibitory mechanism. Cases with attacks of unconsciousness and often epileptiform seizures are called the Stokes-Adams type, and are reviewed in a paper of Clarence Quinan.[11] This condition occurs generally in males over 50, the bradycardia may be either paroxysmal or regular, vaso-motor changes, flushing, and blanching may precede or follow attacks, which are easily brought on by excitement. The venous pulsations may be much more frequent than the apex beat, showing a block between the auricle and ventricle. Changes have been found occasionally in the arteries at the base of the brain, frequently in the coronary vessels, and in the peripheral branches of the vagus. The heart may be normal in size, but it often shows patches of fatty or fibrous myocarditis. Quinan's patient was a baker, aged 72, who had attacks for five months, and often many in a day. The pulse ceased entirely at the wrist during an attack, consciousness was lost, tonic and clonic contractions followed. No movement of the heart could be detected for five to 15 seconds. The pulse between the attacks was from 30 to 40. Any slight excitement or noise or raising the arm above the head would bring on an attack. Under iodide of potash he lost the attacks, but two years later the pulse was only 38. It was found that the blood-pressure was constantly very high, 170–250 mm. The arterial trunks were fairly elastic. Exercise raised the pressure, but did not increase the pulse rate materially. The quantity and gravity of the urine was normal, and the left ventricle was not hypertrophied. How did this feebly acting heart cope with this huge pressure? The writer suggests that the theory of the driving power of the arteries and capillaries (Hasebroek) as a secondary motor mechanism may in some degree explain this.

**Infective Endocarditis.**—H. French [12] points out that we are apt to think that there are only two varieties, the pyæmic and the typhoid, whereas the majority belong to what Dreschfeld calls the cardiac type. In this form there may have been previous rheumatism; then follows later on failing compensation. "Fungating endocarditis" is grafted on to a simple form and a long and varying struggle goes on between the invaders and the organism with an uncertain final result. In the pyæmic and typhoid forms the bacteriology is more or less known; in simple rheumatic endocarditis, Poynton and Payne declare diplococcus rheumaticus is present, but in the cardiac or slower form of infective endocarditis, it is uncertain whether this organism is the active cause, or whether a streptococcal or some similar infection is added. A prolonged irregular pyrexia is not always a sign of pyæmic endocarditis, and may at times end in complete recovery, and other cases of very long duration with multiple emboli also do so, though clearly of a fungating type. Still these two symptoms, as also splenic enlargement and progressive anæmia, count against the heart's condition being due to old fibrosis of the valves alone. Thus there is no hard and fast line between simple and infective endocarditis. The progress and termination of many of the cases is most uncertain. Also since we find that in old cases of valvular disease the natural temperature is often persistently subnormal, a rise in such patients to the normal may be really in them pyrexia and the sign of septic trouble. Withington [13] reports a recovery from malignant endocarditis due to gonorrhœal endocarditis. There were irregular pyrexia, sweating, cough, phlebitis, and pulmonary consolidation, but after a month's illness, the patient made a steady recovery. Barrows [14] describes a case of septicæmia, where streptococci were present, which recovered after two injections of formalin (1 in 5,000) had been given intravenously. Other cases are, however, noted in which no result followed. G. T. Harrison also reports a recovery after intravenous injections of collargol. P. W. Latham [15] holds the view that the value of perchloride of iron in erysipelas, scarlatina, as a local application in diphtheria and in various forms of pyæmia is due to the free chlorine present. Thus it has been given in erysipelas with great success in 20 minim doses every two hours, but the writer thinks that the good results can be obtained with smaller doses or with the addition of doses of chlorine water. To make the latter, Sir Thomas Watson added a drachm of strong HCl. to 20 grains of chlorate of potash in a strong pint bottle. After the violent action had ceased, 20 oz. of water were gradually poured in and the liquid shaken up to absorb the gas. This has been used with great advantage in many forms of septicæmia. Other writers have pointed out that nature employs chlorine in a similar way, as shown by the chlorine metabolism in infective fevers.

[10] Brit. Med. Jour., Oct. 8. [11] Amer. Jour. Med. Sci., Sept. [12] Practit., Dec. [13] Lancet, Sept. 17. [14] Dublin Jour. Med. Sci., Nov. [15] Lancet, Nov. 19.

